# Anti-Proliferative Properties and Proapoptotic Function of New CB2 Selective Cannabinoid Receptor Agonist in Jurkat Leukemia Cells

**DOI:** 10.3390/ijms19071958

**Published:** 2018-07-04

**Authors:** Antonella Capozzi, Vincenzo Mattei, Stefano Martellucci, Valeria Manganelli, Giuseppe Saccomanni, Tina Garofalo, Maurizio Sorice, Clementina Manera, Roberta Misasi

**Affiliations:** 1 Department of Experimental Medicine, University of Rome “La Sapienza”, 00161 Rome, Italy; antonella.capozzi@uniroma1.it (A.C.); s.martellucci@sabinauniversitas.it (S.M.); valeria.manganelli@uniroma1.it (V.M.); tina.garofalo@uniroma1.it (T.G.); maurizio.sorice@uniroma1.it (M.S.); 2 Laboratory of Experimental Medicine and Environmental Pathology, “Sabina Universitas”, 02100 Rieti, Italy; v.mattei@sabinauniversitas.it; 3 Department of Pharmacy, University of Pisa, 56126 Pisa, Italy; giuseppe.saccomanni@farm.unipi.it

**Keywords:** Jurkat leukemia cells, cannabinoid, CB2 receptor, Annexin V, apoptosis, mitochondrial membrane potential

## Abstract

Several studies demonstrated that cannabinoids reduce tumor growth, inhibit angiogenesis, and decrease cancer cell migration. As these molecules are well tolerated, it would be interesting to investigate the potential benefit of newly synthesized compounds, binding cannabinoid receptors (CBRs). In this study, we describe the synthesis and biological effect of 2-oxo-1,8-naphthyridine-3-carboxamide derivative LV50, a new compound with high CB2 receptor (CB2R) affinity. We demonstrated that it decreases viability of Jurkat leukemia cells, evaluated by Trypan Blue and 3-(4,5-dimethylthiazol-2-yl)-2,5-diphenyltetrazolium bromide (MTT), but mainly induces a proapoptotic effect. We observed an increase of a hypodiploid peak by propidium iodide staining and changes in nuclear morphology by Hoechst 33258. These data were confirmed by a significant increase of Annexin V staining, cleavage of the nuclear enzyme poly(ADP-ribose)-polymerase (PARP), and caspases activation. In addition, in order to exclude that LV50 non-specifically triggers death of all normal leukocytes, we tested the new compound on normal peripheral blood lymphocytes, excluding the idea of general cytotoxicity. To characterize the involvement of CB2R in the anti-proliferative and proapoptotic effect of LV50, cells were pretreated with a specific CB2R antagonist and the obtained data showed reverse results. Thus, we suggest a link between inhibition of cell survival and proapoptotic activity of the new compound that elicits this effect as selective CB2R agonist.

## 1. Introduction

Cannabinoid receptors (CBRs) are specific targets for endogenous and exogenous cannabinoids. Among different CBRs, CB2R is almost exclusively expressed in peripheral cells and tissues, derived from the immune system [1], and is unrelated to cannabinoid psychoactivity. In particular, CB1R and CB2R levels are exclusively upregulated in different cancer cells without necessarily being expressed in the tissue type of origin [2,3,4,5].

Behavioral, electrophysiological, and neurochemical studies support a role for CB2R activation in modulating inflammatory nociception, however, beside the palliative actions of cannabinoids, various in vitro studies and animal models have shown that activation of the CB2R induces apoptosis or cell cycle arrest and inhibits neo-angiogenesis, thus inhibiting tumor growth [6,7,8,9,10].

On this regard, it is well known that survival signaling pathways including extracellular signal-regulated kinase (ERK) [11], c-Jun-NH2-kinase [12], p38 mitogen-activated protein kinase (MAPK) [13], and the ceramide pathway [14] can be regulated through CBR activation. The cell cycle arrest is followed by apoptotic death and activation of the transcription factor JunD is essential for these actions [15,16].

In the last decades, several findings indicate that selective CB2R ligands could be promising drugs for treating several diseases [1,17,18,19,20,21,22,23], without psychoactive effects [24]. 

Within a research program to identify CB2R selective ligands, a series of 1,8-naphthyridin-2(1*H*)-on-3-carboxamides was previously reported as CB2R agonists with high affinity and selectivity [25,26,27]. Some of them showed interesting pharmacological properties, such as inhibitory action on immunological human basophil activation and a medium level of intestinal absorption and blood brain barrier permeability [28,29]. Moreover, CB2R dependent anti-proliferative effect in several cancer cell lines was reported for some 2-oxo-1,8-naphthyridine-3-carboxamide derivatives [30], although the precise mechanism of this effect has not been completely investigated yet.

Several molecular mechanisms underlying the cannabinoid-induced cell death have been described. Among these, the events that precede activation of caspases and trigger the executive phase of programmed cell death are included [31]. In fact, changes in the mitochondrial membrane, a potential consequence of loss of integrity of the outer mitochondrial membrane and release of apoptogenic proteins, have been described after synthetic cannabinoid exposure in rat C6 glioma cells [31]. Additionally, a key role for CB2R as potential target in apoptosis induction in malignancies of the immune system has been proposed. This evidence suggests the consideration of CB2R agonists as novel pharmacological anti-cancer agents for tumors of immune origin, selectively [32].

In the present study, we describe the synthesis and biological effect of the new 2-oxo-1,8-naphthyridine derivative LV50 with high CB2R affinity and selectivity. This compound differs from those previously studied [27] for the substituent in position 1 of the central nucleus. We demonstrated that this compound decreases cell viability of the Jurkat leukemia cell line, with a proapoptotic effect higher than that of similar compounds, CB91, LV58, and LV62 previously reported in literature [27]. It is known that Jurkat cells expressed significant levels of CB2R mRNA and low levels of CB1R mRNA [32]. In the present study, we describe an apoptotic pathway elicited by this novel CB2R ligand. Thus, we may suggest a link between inhibition of cell survival and proapoptotic activity of the new compound that elicits this effect as a cannabinoid agonist, suggesting its possible use in anti-cancer therapeutic protocols, in combination with classical anti-neoplastic therapies.

## 2. Results

### 2.1. CB1R and CB2R Affinity

The binding affinities (K_i_ values) of LV50 were evaluated by competitive radioligand displacement assays against the human CB1R and CB2R using [^3^H]CP-55,940 as radioligand for both receptors [26]. The results are summarized in Table 1 with the K_i_ values of the previously reported 2-oxo-1,8-naphthyridine-3-carboxamide derivatives, CB91, LV58, and LV62 [26,27]. The results indicate that LV50 displays excellent affinities for CB2R and low affinities at CB1R, so this compound behaves similarly to previously studied compounds CB91, LV58, and LV62 [26,27]. Moreover, based on our previous studies on 2-oxo-1,8-naphthyridine-3-carboxamide derivatives [27], it is reasonable to assume that LV50 behaves as a CB2R agonist. Indeed, it was previously demonstrated that 2-oxo-1,8-naphthyridine-3-carboxamide derivatives unsubstituted at position C-6 of naphthyridine nucleus, such as LV50, showed a CB2R agonist behavior [27].

### 2.2. CB2R Expression

**Figure 1:**
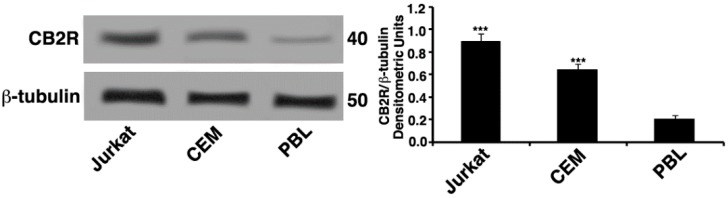
CB2R expression. Whole cell extracts of Jurkat, lymphoblastoid T cell line (CEM), and peripheral blood lymphocytes (PBL) were analyzed by Western blot to detect cannabinoid receptor 2 (CB2R) levels using antibodies specific for CB2R protein. The loading control was evaluated using anti-tubulin mAb. Densitometric CB2R/β-tubulin ratio is shown. The results are represented as the mean ± SD from three independent experiments. Statistical analysis indicated: *** *p* < 0.001 versus PBL cells.

The expression of CB2R was determined by Western blot. Whole cell lysates of Jurkat, lymphoblastoid T cell line (CEM) and peripheral blood lymphocytes (PBL) were analyzed; the results showed that Jurkat and CEM cells lines expressed significant levels of CB2R protein compared with PBL cells (Figure 1). 

### 2.3. Preliminary Analysis of the Compounds

To select the most active compound, we have performed a preliminary analysis evaluating cell viability and proliferation. Jurkat cells were treated with CB91, LV58, LV62, and LV50 (concentration range 0.1–10 μM) for different incubation times (24–72 h) and then analyzed to investigate cell viability [Trypan Blue and 3-(4,5-dimethylthiazol-2-yl)-2,5-diphenyltetrazolium bromide (MTT) assay] and pro-apoptotic effect [propidium iodide (PI) staining]. In addition, a dose-dependent effect of CB91, LV62, and LV58 compounds on cell viability was assessed as shown in the Supplementary Figure S1. The most effective results were obtained at 10 μM concentration (Table 2), indicating LV50 as the most interesting compound deserving further biological activity studies. 

Similar analyses have also been performed in the presence of CB2R antagonist SR144528 (1 μM). The results are shown in Table 3. 

### 2.4. LV50 Reduces Cell Viability

In order to exclude a Jurkat-specific effect or a non-specific cytotoxic effect on normal leukocytes, we tested the dose-dependent effect of LV50 on Jurkat, CEM, and PBL cell viability by using Trypan Blue assay.

**Figure 2:**
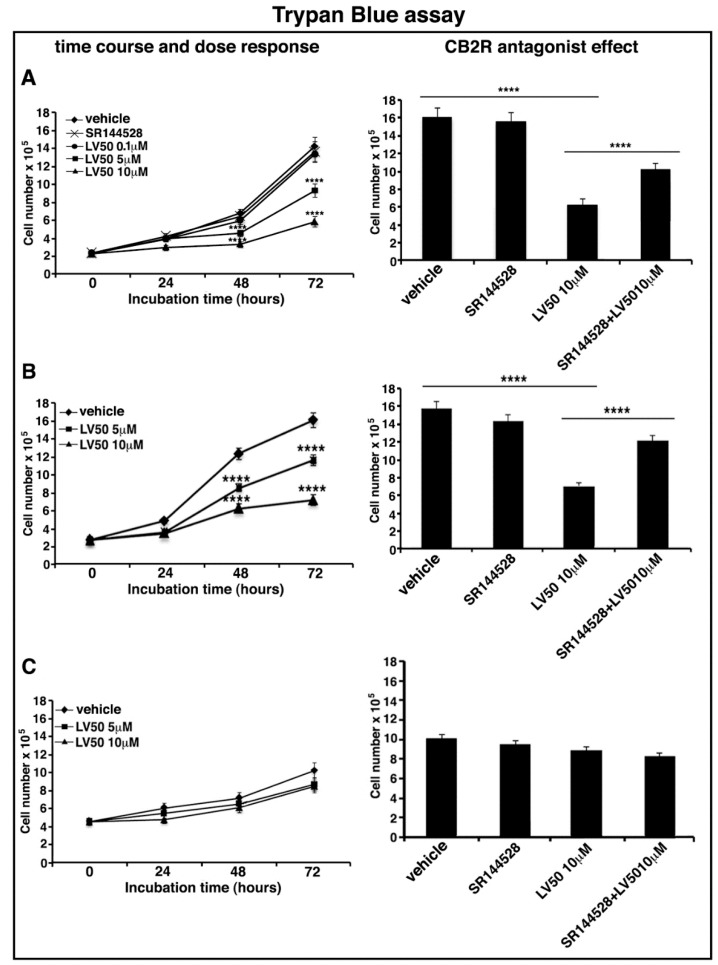

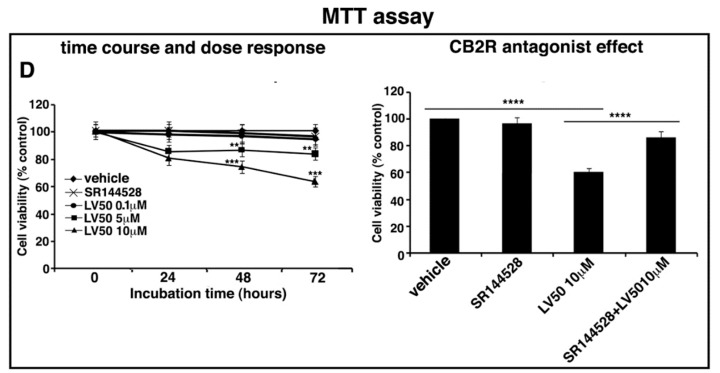
LV50 decreases cell viability and inhibits cell proliferation. Jurkat, CEM, and PBL cells were cultured with different concentrations of LV50 for 24, 48, and 72 h. The number of viable cells was determined by Trypan Blue exclusion test. (**A**, **left panel**) Jurkat cells, data are reported as the mean ± SD among ten independent experiments. Statistical analysis indicated: **** *p* < 0.0001 versus vehicle. (**A**, **right panel**) Jurkat cells were pretreated with selective antagonist for CB2R (SR144528, 1 μM), exposed to LV50 for 72 h and then analyzed for cell viability. Statistical analysis indicated: **** *p* < 0.0001 versus vehicle; **** *p* < 0.0001 versus pretreated with SR144528. (**B**, **left panel**) CEM cells, data are reported as the mean ± SD among ten independent experiments. Statistical analysis indicated: **** *p* < 0.0001 versus vehicle. (**B**, **right panel**) CEM cells were pretreated with selective antagonist for CB2R (SR144528, 1 μM), exposed to LV50 for 72 h and then analyzed for cell viability. Statistical analysis indicated: **** *p* < 0.0001 versus vehicle; **** *p* < 0.0001 versus pretreated with SR144528. (**C**) PBL cells, data are reported as the mean ± SD among ten independent experiments. Statistical analysis indicated: LV50 10 μM versus vehicle NS (not significant). (**D**, **left panel**) Cell proliferation was measured by 3-(4,5-dimethylthiazol-2-yl)-2,5-diphenyltetrazolium bromide (MTT) assay in Jurkat cells. The results represent the mean ± SD of five independent experiments performed in triplicate and represent cell viability as a percentage of untreated control cells. Statistical analysis indicated: ** *p* < 0.01 versus vehicle; *** *p* < 0.001 versus vehicle. (**D**, **right panel**) Jurkat cells were pretreated with selective antagonist for CB2R (SR144528, 1 μM), exposed to LV50 for 72 h and then analyzed for proliferation. Statistical analysis indicated: **** *p* < 0.0001 versus vehicle; **** *p* < 0.0001 versus pretreated with SR144528.

In Jurkat and CEM cells, the results show a reduction of cell viability in the samples treated with 5 μM and 10 μM concentrations of the compound. The number of viable cells is significantly reduced at 10 μM concentration after 72 h of LV50 treatment, compared with control vehicle-treated cells (left panel of Figure 2A,B). No significant cytotoxicity can be observed in PBL cells after LV50 treatment (Figure 2C, left panel).

Furthermore, we evaluated the anti-proliferative dose-dependent effect of LV50 on Jurkat cells, determined by MTT assay at various time points. We observed an anti-proliferative effect proportional to the rate of MTT cleavage reaction in treated samples in a dose- and time-dependent manner, as compared with vehicle-treated cells (Figure 2D, left panel). 

Moreover, in order to demonstrate that molecular mechanism of the new compound may involve CB2R, we performed the experiments in the presence of a selective antagonist for CB2R, SR144528 (1 μM). Figure 2A (right panel), Figure 2B (right panel), and Figure 2D (right panel) showed that cell pretreatment with CB2R antagonist partially reversed the cytotoxic and anti-proliferative effect induced by LV50. 

Instead, no significant reduction of cell viability or proliferation was observed in cells treated with CB2R antagonist SR144528 alone (left panel of Figure 2A,D).

We observed similar results in CEM cells, whereas no significant effect in PBL cells was observed (data not shown).

### 2.5. Pro-Apoptotic Activity of LV50

#### 2.5.1. LV50 Increases the Percentage of Cells in Apoptotic Sub-G1 Population and Nuclear Morphological Changes

**Figure 3:**
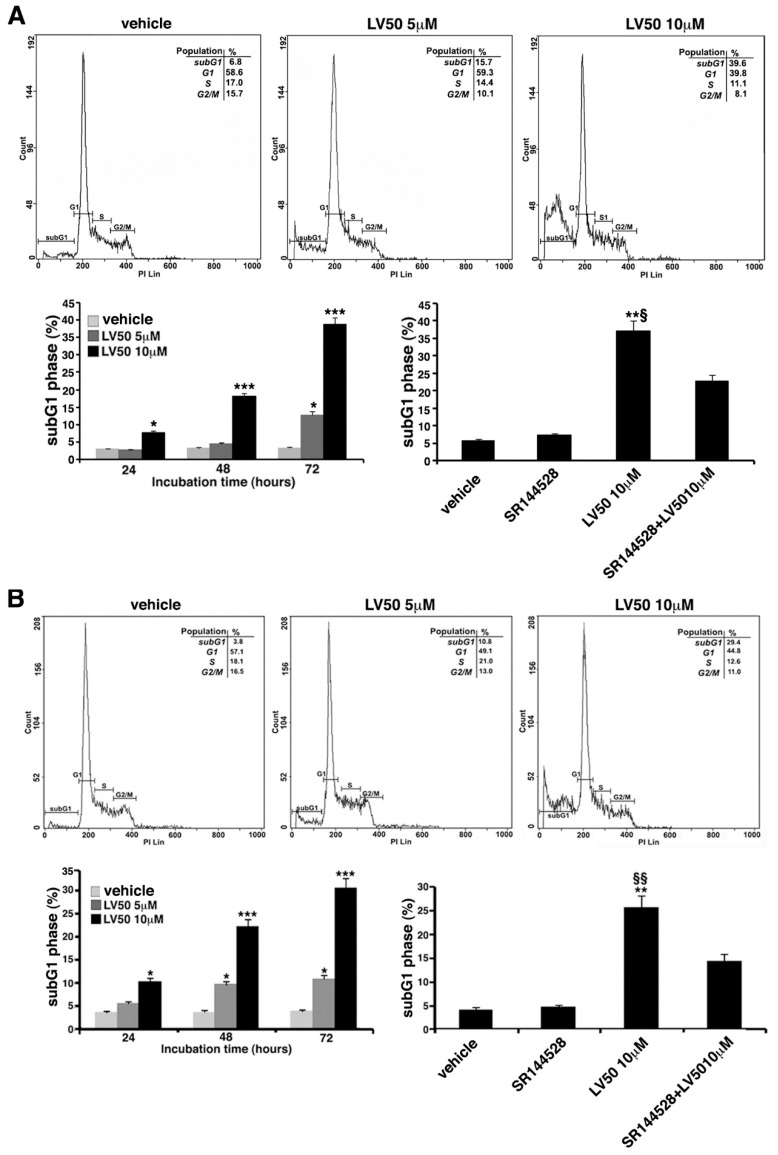

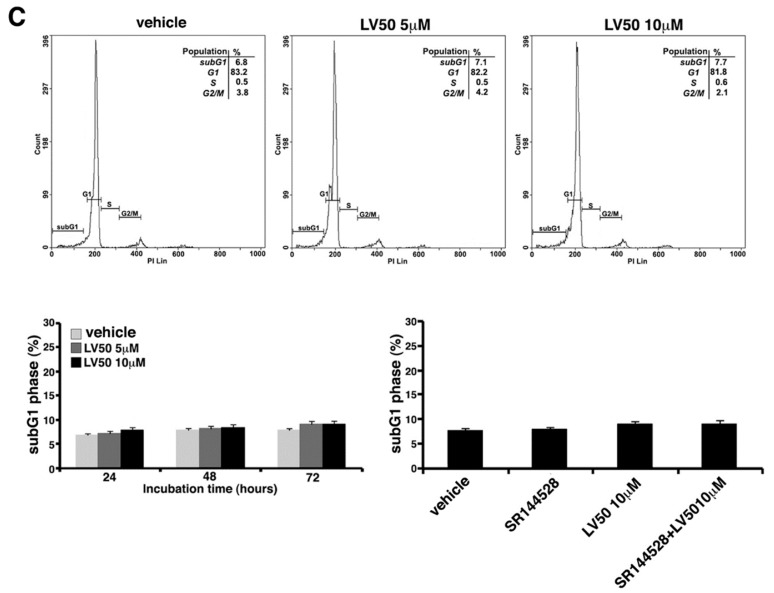
Effect of LV50 on nuclear fragmentation. Jurkat, CEM, and PBL cells incubated with 5 μM or 10 μM LV50 for 72 h were analyzed by flow cytometric analysis after staining with propidium iodide. Histograms show cell cycle profiles and hypodiploid sub-G1 peak indicates typical DNA fragmentation that defines apoptosis. The percentage of cells in each phase of cell cycle is summarized below the histograms. Vehicle-treated or cells incubated with 5 μM or 10 μM LV50 were analyzed at three incubation times (24, 48, and 72 h). (**A**, **left panel**) Jurkat cells, results represent mean ± SD of three independent experiments. Statistical analysis indicated: * *p* < 0.05 versus vehicle; *** *p* < 0.001 versus vehicle. (**A**, **right panel**) In Jurkat cells, Propidium Iodide staining was performed in the presence or absence of the selective CB2R antagonist (1 μM). The results represent mean ± SD of three independent experiments. Statistical analysis indicated: ** *p* < 0.01 versus vehicle; § *p* < 0.05 versus pretreated with SR144528. (**B**, **left panel**) CEM cell, results represent mean ± SD of three independent experiments. Statistical analysis indicated: * *p* < 0.05 versus vehicle; *** *p* < 0.001 versus vehicle. (**B**, **right panel**) In CEM cells, propidium iodide staining was performed in the presence or absence of the selective CB2R antagonist (1 μM). The results represent mean ± SD of three independent experiments. Statistical analysis indicated: ** *p* < 0.01 versus vehicle; §§ *p* < 0.01 versus pretreated with SR144528. (**C**) PBL cells, results represent mean ± SD of three independent experiments. Statistical analysis indicated: LV50 10 μM versus vehicle NS (not significant).

Cell cycle and DNA content were measured in Jurkat, CEM, and PBL cells, by cytofluorimetric analysis using PI staining. However, the main result is an evident sub-G1 peak in LV50 treated cells that identifies DNA fragmentation as typical nuclear changes that define apoptosis (Figure 3A,B). We found a significant increase in sub-G1 phase when cells were treated with LV50 10 μM for 48 or 72 h (left panel of Figure 3A,B). In PBL cells treated with LV50, we obtained no significant pro-apoptotic effect (Figure 3C). Pretreatment with SR144528 (1 μM) selective antagonist for CB2R showed a modulation of LV50 induced cytotoxic effect, suggesting a role for CB2R in this molecular mechanism (right panel of Figure 3A,B).

#### 2.5.2. Pro-Apoptotic Activity of LV50 Revealed by Hoechst 33258, Annexin V Staining and Caspases Activation

**Figure 4:**
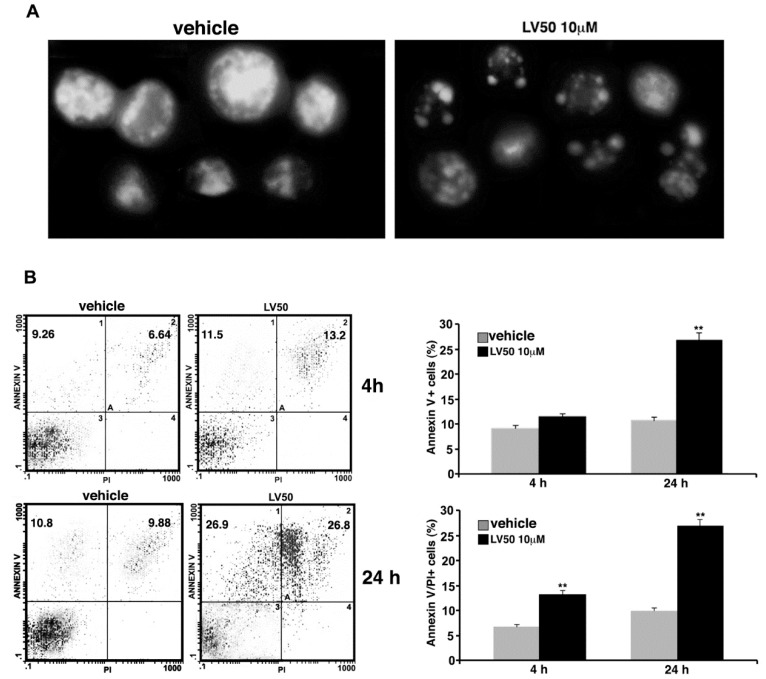
Pro-apoptotic activity of LV50. (**A**) Morphological analysis of Jurkat nuclei stained with Hoechst 33258 after incubation with 10 μM LV50 for 72 h. Vehicle-treated cells are uniformly stained and the nuclei are intact, indicating that cells are viable; treatment causes nuclear fragmentation and condensation. A representative example of three independent experiments. (**B**, **left panel**) Analysis of apoptosis of Jurkat cells (treated with 10 μM LV50 for 4 and 24 h) by biparametric flow cytometry analysis after double staining with fluorescein isothiocyanate (FITC) Annexin V/PI staining. Numbers represent the percentage of Annexin V-single positive cells (early apoptosis, upper left quadrant) or Annexin V/propidium iodide (PI)-double positive cells (late apoptosis, upper right quadrant). (**B**, **right panel**) The results shown in bar graphs were obtained from three independent experiments and are reported as mean ± SD. Histograms show the percentages of single Annexin V and Annexin V/PI-double positive cells. ** *p* < 0.01 versus vehicle.

Data obtained on the proapoptotic activity of LV50 were confirmed in Jurkat cells, by a morphological approach using Hoechst 33258. Nuclei of cells treated with the compound appeared fragmented with a higher chromatin condensation, compared with vehicle-treated cells where we observed intact nuclei (Figure 4A). 

Cell apoptosis was further analyzed by flow cytometry using Annexin V/PI staining. In cells treated with LV50 for 4 h, we observed a significant increase of Annexin V/PI positive cells (13.2 ± 0.8%), as compared with the vehicle (*p* < 0.01) (Figure 4B, upper panel). At longer exposure times (24 h), we observed a higher Annexin V staining at the cell surface in a significant percentage (26.8 ± 1.4) of cells (Annexin V/PI positive), with a further increase of percentage of PI positive cells, typical of the late phase of apoptosis (Figure 4A, bottom panel). Statistical analysis was performed on data obtained from three independent experiments (Figure 4). 

**Figure 5:**
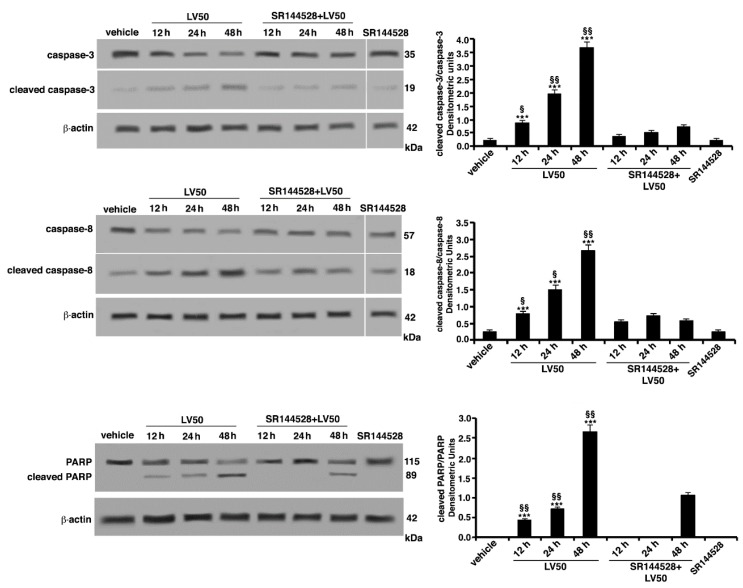
LV50 induce activation of caspase-3, caspase-8, and poly(ADP-ribose)-polymerase (PARP) as indicated by detection of cleaved proteins after Western blot analysis. Jurkat cells were treated with the compound, or alternatively, pretreated with CB2R antagonist SR144528 (1 μM) and then incubated with LV50 10 μM for indicated incubation times. Whole cell extracts were analyzed by antibodies specific for uncleaved caspase-3, cleaved caspase-3, uncleaved caspase-8, cleaved caspase-8, and PARP. The loading control was evaluated using anti-actin mAb. Densitometric cleaved caspase-3/uncleaved caspase-3, cleaved caspase-8/uncleaved caspase-8, and cleaved PARP/uncleaved PARP ratios are shown. The results are represented as the mean ± SD from three independent experiments. Statistical analysis indicated: *** *p* < 0.001 versus vehicle; §§ *p* < 0.01 versus pretreated with SR144528; § *p* < 0.05 versus pretreated with SR144528.

In parallel experiments, the expression of caspases and poly(ADP-ribose)-polymerase (PARP) protein was evaluated by Western blot analysis. LV50 treatment induced a significant increase, in a time-dependent manner, of the active cleaved form of caspase-3 expression (Figure 5), a key molecule for induction of caspases activation. Among initiator caspases, we analyzed the expression of the cleaved form of caspase-8, which significantly appeared with a higher expression in the samples treated with LV50, as compared with the vehicle-treated sample (Figure 5). As a consequence of their proteolytic functions, caspase activation resulted in the cleavage of the downstream substrates, including the nuclear enzyme PARP engaged in DNA repair. In fact, we observed that the level of cleaved PARP protein increased with the time of incubation after LV50 treatment. These findings suggest that LV50-induced Jurkat cell death involves caspase activation. Furthermore, cleaved caspases and cleaved PARP expression significantly decreased when cells were pretreated with the selective CB2R antagonist SR144528 (1 μM), supporting the view of an involvement of CB2R in the LV50 proapoptotic activity (Figure 5). On the contrary, no significant increase of cleaved forms of caspase-3, caspase-8, or PARP was evident when cells were treated with CB2R antagonist SR144528 alone.

### 2.6. Molecular Involvement during Apoptotic Execution

**Figure 6:**
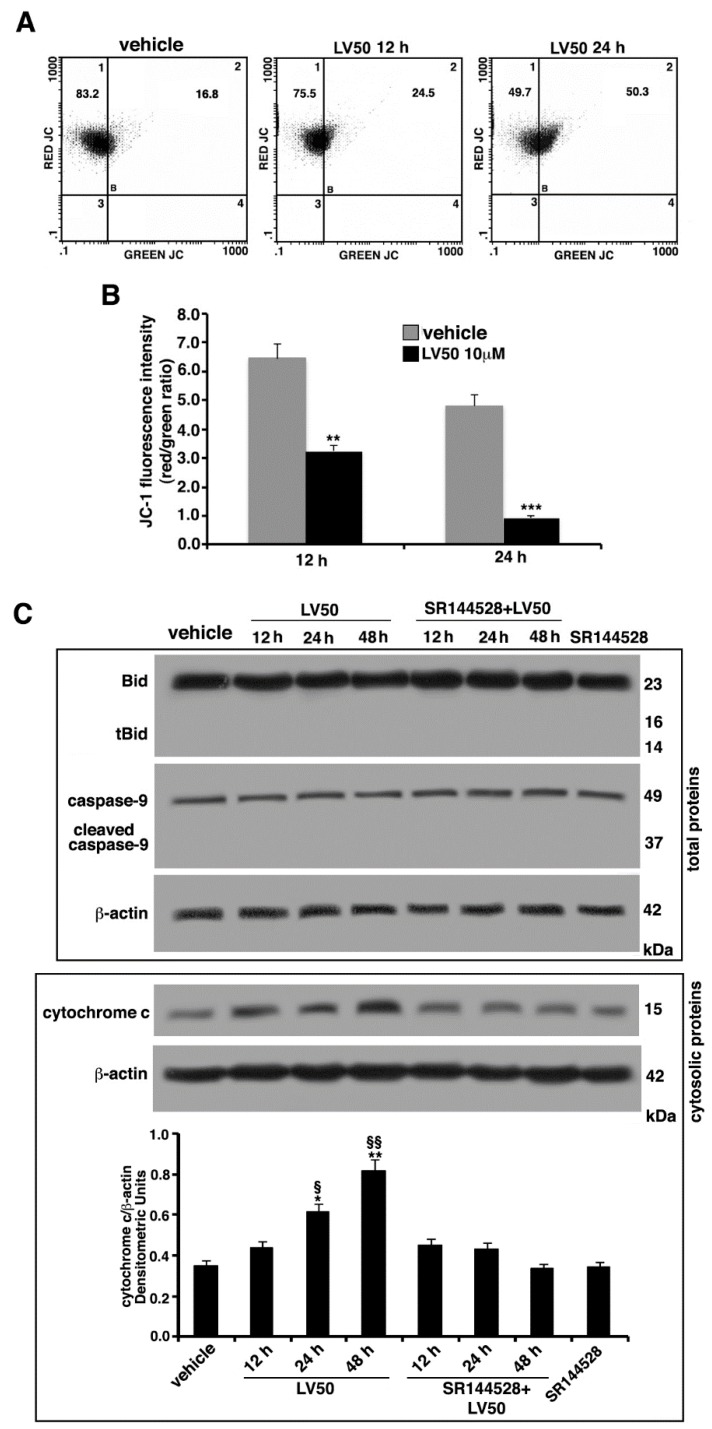
Molecular involvement during apoptotic execution. (**A**) Analysis of mitochondrial membrane permeabilization (MMP) of Jurkat cells (treated with 10 μM LV50 for 12 and 24 h) by biparametric flow cytometry after staining with 5,5′,6,6′tetraethylbenzimidazolylcarbocyanine iodide (JC-1). Numbers reported represent the percentage of cells with depolarized mitochondria (green florescence, upper right quadrant). (**B**) Bar graphs show results obtained from three independent experiments and reported as mean ± SD. Histograms show JC-1 fluorescence intensity red/green ratio indicating an increase of green fluorescence in treated cells, represented as a lower ratio, compared with vehicle-treated cells. ** *p* < 0.01 (12 h); *** *p* < 0.001 (24 h). (**C**) Jurkat cells were treated with the compound, or alternatively, pretreated with CB2R antagonist SR144528 (1 μM) and then incubated with LV50 for indicated incubation times. Whole cell extracts were analyzed by Western blot using antibodies specific for Bid/t-Bid and cleaved caspase-9/uncleaved caspase-9. Furthermore, cytosolic fraction was analyzed by Western blot for cytochrome c release. The results are represented as the mean ± SD from three independent experiments. Statistical analysis indicated: * *p* < 0.05 versus vehicle; § *p* < 0.05 versus pretreated with SR144528; ** *p* < 0.01 versus vehicle; §§ *p* < 0.01 versus pretreated with SR144528.

Changes in the mitochondrial membrane permeabilization (MMP) (ΔΨ) are usually associated with Apoptosis [33]. Thus, MMP alterations were assessed by flow cytometry, using the fluorescent probe 5,5′,6,6′tetraethylbenzimidazolylcarbocyanine iodide (JC-1), a cationic carbocyanine dye that accumulates in mitochondria. JC-1 forms monomers (low MMP, green fluorescence) or aggregates (high MMP, red fluorescence). As shown in Figure 6, vehicle-treated cells exhibited intensive red fluorescence, consistent with the formation of JC-1 J-aggregates. Conversely, LV50 exposure induced an evident reduction of red fluorescence and enhanced green fluorescence intensity, indicative of a change in the ΔΨ, in the populations induced to undergo apoptosis (Figure 6A). In fact, cells treated with the compound, compared with the control, appeared to display a depolarization of the mitochondrial membrane in a time-dependent manner. However, the two fluorescences (green and red) do not appear exactly inversely proportional, in fact, the increase in green fluorescence was not accompanied by a proportional decrease in red fluorescence. This is a limit of the specific cytofluorimetric analysis of JC-1, so much so that the ratio between the two fluorescences is indicated. Statistical significance of these data was confirmed by analyzing results obtained from three independent experiments. Values of the mean fluorescence ratio red/green (Figure 6B) significantly decreased in LV50-treated cells compared with the vehicle-treated cells, after 12 h and 24 h of treatment (*p* < 0.01 and *p* < 0.001, respectively). 

In order to better characterize the apoptotic pathway and the proteins addressing apoptotic execution, we analyzed by Western blot the Bcl2 family member Bid, truncated Bid (t-Bid), caspase-9, and cytochrome c, following LV50 treatment. As shown in Figure 6C, low levels of t-Bid and cleaved caspase-9 were detected. Moreover, a significant increase of cytochrome c was revealed (Figure 6C), suggesting that both apoptotic pathways may be involved, although in this case, the extrinsic type appears to be predominant. 

## 3. Discussion

The possibility to use CB2R as a key molecule to convey any adjuvant drugs of classical chemotherapy has for some time been a suggestive proposal of the scientific world [34]. 

In fact, CB2R has been found in many types of cancer cells, including human leukemia and lymphoma cell lines. As reported, activation of the CB2R induces apoptosis or cell cycle arrest and inhibits neo-angiogenesis, thus inhibiting tumor growth and progression [8,9].

Among the involved molecular mechanisms, much has been studied both for the signal transduction and the apoptotic pathway [13,31]. In this work, we demonstrate the anti-proliferative and proapoptotic activity of a newly synthesized compound, LV50, with a marked CB2R affinity and selectivity.

In particular, we show that LV50, a new 2-oxo-1,8-naphthyridine derivative, was capable of eliciting cytotoxic effects in cancer cells, such as Jurkat and CEM lymphoblastoid cells, but not in peripheral blood lymphocytes, thus excluding the idea of general cytotoxicity or cell line specific effect. These data were confirmed by evaluating the percentage of proliferating cells stained by MTT assay, which clearly displayed a significant anti-proliferative effect in a dose- and time-dependent manner. In addition, LV50 appears to be a good apoptosis inducer in leukemia cells, as shown by flow cytometric analysis of PI stained cells.

The regulation of the apoptotic mechanism is very complex from a molecular point of view. However, this has a positive implication, because of the high number of molecular actors in the apoptotic pathway, which offer ample opportunities for interaction with new therapeutic strategies [31,35]. This is the reason we decided to focus on molecules belonging to apoptotic machinery. 

The pharmacological treatment of cancer has, as a general rule, the objective of improving the clinical response to treatments, using strategies to weaken the proliferation of cancer cells, control unwanted cellular toxicity, and overcome the intrinsic resistance to the drug. Most of the chemotherapeutic agents have been identified by virtue of their cytotoxicity against tumor cell lines. On this purpose, a further important point is represented by the induction of apoptosis, which represents an important goal for anti-tumor treatment strategies [7,8,36]. Cannabinoids effects in the cell cycle modulation and in the signaling pathways are different and may depend on the type of cancer cells. Although there is evidence that some cannabinoids induce anti-proliferative effects on cancer cells in vitro, at the same time, some evidences suggest that effects may depend on the context of the disease, with differential effects observed in different types of cancer [7,8,9,10,37,38,39,40].

An increase in apoptotic cells was observed after treatment of Jurkat leukemia cells with 10 μM concentration of the LV50 compound. Indeed, at shorter time (4–24 h) of LV50 exposure, the cells displayed a clear positivity of the Annexin V test, indicating the early stages of apoptotic pathway. This effect became more meaningful at longer exposure times, studied with PI staining and cytofluorimetric analysis, with the increase of sub-G1 peak after 72 h, which was evident not only in terms of percentage, but also in the morphology of the sub-G1 peak, which identifies DNA fragmentation as typical nuclear changes that define apoptosis. Apoptotic data were confirmed by a morphological approach using Hoechst 33258. Indeed, nuclei of cells treated with LV50 showed the typical fragmentation with a higher chromatin condensation. Moreover, the increase of the active cleaved form of caspase-3 and caspase-8 in the samples treated with LV50, compared with vehicle-treated cells, as well as the cytochrome c release and the depolarization of the mitochondrial membrane in a time-dependent manner after LV50 treatment, indicated both extrinsic and intrinsic apoptotic triggering of the LV50 treatment.

It is interesting to note that the effects of LV50 of this study were partially inhibited by the pretreatment of cells with selective CB2R antagonist SR144528, indicating a clear involvement of CB2R in the signaling pathway of the cytotoxic mechanism triggered by LV50.

Identifying the molecular pathways that trigger apoptosis following the engagement of CBRs is essential to understand how cannabinoids can regulate the growth of immune cells. In 1975, Munson and collaborators [41] were the first to report the anti-proliferative properties of cannabis compounds. Our data, in accordance with the literature, suggest that apoptotic mechanism through CB2R may involve both extrinsic and intrinsic pathways of apoptosis [42]. However, on the basis of our findings, we suggest that, in this case, the involved apoptotic pathway may be predominantly of an extrinsic type.

In conclusion, our study provides new information on the development of a novel class of anti-cancer drugs targeting CB2R.

Although our study is not the first to demonstrate that activation of cannabinoid receptors on transformed T cells triggers apoptosis [32,40,43,44], the data presented in this study provide evidence for the first time of a recently synthesized molecule that, by binding CB2R, triggers an apoptotic pathway, involving new insights into the functional roles of cannabinoid receptors.

In fact, the use of cannabinoids that activate CB1R is very limited by their side effects [24,45]. In this case, the malignant cells of the immune system express CB2R and this molecule can be targeted by LV50 to induce apoptosis. CBR ligands, in addition to their anti-proliferative effect on cancer cells, exert palliative effects that prevent nausea, vomiting, and pain and stimulate appetite in cancer patients receiving chemotherapy or conventional radiotherapy. Thus, we suggest the possibility of using selected CB2R protein agonists, including LV50, in a new role as anticancer drugs, in particular as part of a mixture of molecules able to target the CBR system in order to improve the antitumor activity of chemotherapy and to soften unwanted iatrogenic side effects, without psychoactive properties.

## 4. Materials and Methods

### 4.1. Synthesis of 1-(4-Chlorobutyl)-1,2-dihydro-N-(4-methylcyclohexyl)-2-oxo-1,8-naphthyridine-3-carboxamide (LV50)

**Scheme 1:**
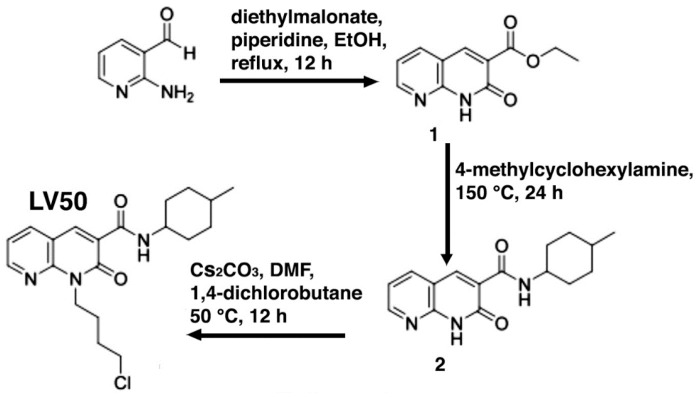
Synthesis of 2-oxo-1,8-naphthyridine-3-carboxamide derivative LV50.

The synthetic route to obtain LV50 is outlined in Scheme 1. The reaction at reflux of 2-aminopyridin-3-carboxaldheyde with diethylmalonate in EtOH and in the presence of piperidine afforded the ethyl ester **1** [26], which was treated with a *cis*/*trans* diastereoisomeric mixture of 4-methylcyclohexylamine in a sealed tube at 150 °C for 24 h, to give the carboxamide **2**. The desired 2-oxo-1,8-naphthyridine-3-carboxamide derivative LV50 was obtained by *N*-alkylation of carboxamide **2** with the 1,4-dichlorobutane in anhydrous *N*,*N*-Dimethylformamide (DMF) and in the presence of cesium carbonate at 50 °C for 12 h.

LV50 was purified by flash chromatography using hexane/ethyl acetate 1:1.2 *v*/*v* as eluent in 30% yield. ^1^H-NMR (CDCl_3_) δ 10.03 and 9.65 (2m, 1H, NH); 8.88 (s, 1H, Ar); 8.74 (m, 1H, Ar); 8.09 (m, 1H, Ar); 7.30 (m, 1H, Ar); 4.63 (m, 2H, CH_2_); 4.26 and 3.93 (2m, 1H, CH); 3.63 (m, 2H, CH_2_); and 1.94–0.92 (m, 16H, 4-methylcyclohexyl+ 2CH_2_). ^13^C-NMR (CDCl_3_) δ 162.73, 161.96, 152.13, 149.86, 141.90, 138.58, 123.41, 119.23, 115.10, 49.03, 45.87, 44.87, 41.22, 34.18, 33.23, 32.25, 31.34, 30.45, 30.24, 30.34, 29.88, 25.78, 22.53, and 21.82.

### 4.2. CB1 and CB2 Receptor Binding Assays 

LV50 was evaluated in CB1R and CB2R binding following the same procedure previously reported for CB91, LV58, and LV62 [26,27]. Briefly, the membranes expressing *h*CB1R or *h*CB2R were incubated with vehicle or LV50 and [^3^H]CP55,940) [46] for 90 min at 30 °C. Non-specific binding was determined in the presence of 100 nM of WIN55212-2 [47]. K_i_ values were calculated by applying the ChengePrusoff equation [48] to the half maximal inhibitory concentration IC_50_ values (obtained by GraphPad) for the displacement of the bound radioligand by increasing LV50 concentration. 

### 4.3. Cell Culture and Treatments

Jurkat leukemia cells and human lymphoblastoid CEM cells (American Type Culture Collection, ATCC, Manassas, VA, USA) were cultured in Roswell Park Memorial Institute medium (RPMI) 1640 medium, containing 10% fetal bovine serum (FBS) supplemented with 2 mM l-glutamine, 100 units/mL penicillin, and 10 mg/mL streptomycin (Sigma-Aldrich, Milan, Italy). Cells were grown under standard conditions at 37 °C in a humified incubator containing 5% CO_2_. Human peripheral blood mononuclear cells were isolated from the blood of healthy donors by Lymphoprep density-gradient centrifugation (Nycomed Pharma, Oslo, Norway). Then, cells were seeded in a flask for 12 h, and we have recovered suspension cells to be used as peripheral blood lymphocytes (PBL) excluding adherent cells.

Derivatives of 1,8-naphthyridin-2(1*H*)-on-3-carboxamide, namely, CB91, LV58, LV62, and LV50, were dissolved in dimethyl sulfoxide (DMSO, Sigma-Aldrich) at the 10-mM stock solution. 

Cells were seeded into a 12-well cell culture plate at the concentration of 2 × 10^5^ cells/mL and incubated for 24 h at 37 °C with 5% CO_2_. Then, cells were treated with different concentrations of compound (0.1–10 μM range) in RPMI medium, containing only 1% FBS avoiding interactions of the compound with cannabinoid receptor ligands and serum proteins. The incubation times used were in the range of 4–72 h.

To investigate CB2R involvement, in some experiments, we pretreated Jurkat cells with a selective CB2R antagonist SR144528 (Tocris Bioscience, Bristol, UK), 1 μM was added 2 h before compound.

We consider vehicle cells without any treatment with only culture medium plus DMSO used as a vehicle to dissolve the compounds.

### 4.4. Western Blot Analysis of CB2R Expression

To prepare whole-cell protein extracts, Jurkat, CEM, and PBL cells were lysed in lysis buffer containing 1% Triton X-100, 10 mM Tris-HCl (pH 7.5), 150 mM NaCl, 5 mM EDTA, 1 mM Na_3_VO_4_, and 75 U of aprotinin. The protein content was analyzed by Bradford assay (Bio-Rad, Segrate, Italy). Equal amounts of whole-extract proteins were subjected to 10% sodium-dodecyl sulphate polyacrylamide gel electrophoresis (SDS-PAGE) before electrophoretic transfer onto polyvinilidene difluoride (PVDF) membranes (Bio-Rad). After blocking with 5% defatted dried milk in tris-buffered saline (TBS), containing 0.05% tween 20, the membranes were incubated overnight with rabbit anti-CB2R (Abcam, Cambridge, UK). This reaction was followed by incubation with secondary antibodies such as horseradish peroxidase (HRP)-conjugated anti-rabbit IgG Abs (Sigma-Aldrich). Immunoreactivity was detected using an enhanced chemiluminescence reaction by the enhanced chemiluminescence ECL Western blotting detection system (Amersham, Buckinghmashire, UK). As a control for loading and purity of preparation, membranes were stripped and reprobed with monoclonal anti-tubulin antibodies (Sigma-Aldrich).

Densitometric scanning analysis was performed by Mac OS X (Apple Computer International, Cupertino, CA, USA), using NIH Image 1.62 software. The density of each band (absolute value) in the same gel was analyzed.

### 4.5. Proliferation and Cell Viability Assays

Vehicle-treated cells or cells incubated with compounds (24, 48, 72 h) were analyzed by Trypan Blue (Sigma-Aldrich) assay to evaluate cell viability and cytotoxic effect [49].

Cell proliferation was evaluated by MTT [3-(4,5-Dimethylthiazol-2-yl)-2,5 diphenyltetrazoliumbromide] (ATCC, Manassas, VA, USA) assay. Briefly, after treatment MTT (10 μL) solution (5 mg/mL) was added to each well. The reaction was allowed to proceed for 4 h at 37 °C. The culture medium was removed and the water insoluble dark blue formazan crystals that formed from MTT cleavage in actively metabolizing cells were dissolved by adding DMSO (200 μL). The absorbance of each well was read at 570 nm and directly correlated with the number of remaining viable cells. All of the samples and related measurements were carried out in triplicate.

### 4.6. Apoptosis Assays

#### 4.6.1. Propidium Iodide Staining

Vehicle-treated cells or cells incubated with compound (24, 48, 72 h) were analyzed for apoptosis by flow cytometry, and PI staining was used to study DNA fragmentation. Briefly, cells were washed twice in phosphate buffered saline (PBS) and fixed in 70% ethanol in PBS for 1 h at 4 °C. Then, cells were washed twice with PBS, resuspended in 0.5 mL PBS, 50 μL RNase A (5 μg/mL) (Sigma-Aldrich), and stained with 0.5 mL of 100 mg/mL propidium iodide (Sigma-Aldrich) in PBS. Cells were incubated for 30 min at room temperature in the dark and analyzed for DNA content and the fluorescence was measured using a EPICS profile cytometer (Coulter Electronics, Brea, CA, USA). 

#### 4.6.2. Hoechst Staining

Cells, without any treatment or incubated with the compound as described above, were also analyzed for apoptosis by morphological analysis. Changes in nuclear morphology were analyzed by DNA-binding fluorescent dye Hoechst 33258 (Sigma-Aldrich). Cells were washed twice with PBS and fixed in 70% ethanol in PBS. After further washes with PBS, vital dye Hoechst 33258 was used at a final concentration of 1 μM. A minimum of 2 × 10^5^ cells were analyzed per sample, employing a Leitz DMRB (Leica Microsystems Wetzlar GmbH, Wetzlar, Germany) inverted fluorescence photomicroscope (320 nm UV excitation) and photographed under fluorescent light. Nuclei of cells characterized by marked chromatin condensation were scored as apoptotic.

#### 4.6.3. Annexin V Assay

Alternatively, in cells treated with 10 μM LV50 for 4 and 24 h, apoptosis was evaluated by flow cytometry using a FITC (fluorescein isothiocyanate) Annexin V apoptosis detection kit (BD Bioscience, San Diego, CA, USA). Protocol provides for a double staining with FITC Annexin V to identify apoptotic cells and PI to distinguish viable cells from nonviable cells. This assay allows discrimination among early apoptotic (single Annexin V positive), late apoptotic (double Annexin V/PI positive), and necrotic cells (single PI positive). The fluorescence was measured using an EPICS profile cytometer (Coulter Electronics). 

### 4.7. Western Blot Analysis of Caspase and PARP Proteins

Cells were treated with 10 μM LV50 for 12–48 h. To prepare whole-cell protein extracts, Jurkat cells without any treatment or cells treated with LV50 were lysed in lysis buffer (as above). After SDS-PAGE and electrophoretic transfer onto PVDF, the membranes were incubated overnight with rabbit anti-caspase-3, rabbit anti-cleaved caspase-3, rabbit anti-caspase-8, mouse anti-cleaved caspase-8, anti-caspase-9, and rabbit anti-PARP (Cell Signaling, Danvers, MA, USA). These reactions were followed by incubation with secondary antibodies such as horseradish peroxidase (HRP)-conjugated anti-rabbit IgG Abs and anti-mouse IgG (Sigma-Aldrich). Immunoreactivity was detected using the ECL Western blotting detection system (Amersham). As a control for loading and purity of preparation, membranes were stripped and reprobed with monoclonal anti-actin antibodies (Sigma-Aldrich).

Densitometric scanning analysis was performed by Mac OS X (Apple Computer International), using NIH Image 1.62 software. The density of each band (absolute value) in the same gel was analyzed.

### 4.8. Analysis of Mitochondrial Membrane Potential Changes (ΔΨ)

The MMP of vehicle-treated cells or cells incubated with LV50 (12 h and 24 h) was studied by flow cytometry using 5,5′,6,6′tetraethylbenzimidazolylcarbocyanine iodide (JC-1) (Molecular Probes, Eugene, OR, USA) [50]. Cells were incubated with 10 μM JC-1 for 15 min at 37 °C in the dark. JC-1 is a sensitive marker for MMP, which is a cationic carbocyanine dye able to selectively enter the mitochondria. The dye exists in a monomeric form, at low concentrations, yielding green fluorescence, but at higher concentrations (as a result of high mitochondrial membrane potential), it can form J-aggregates that exhibit a broad excitation spectrum (red fluorescence). MMP collapse is typical of apoptotic cells with a decrease in the aggregate fluorescent count resulting in a return of the dye to its monomeric, green fluorescent form. The fluorescence was measured using an EPICS profile cytometer (Coulter Electronics). 

### 4.9. Analysis of t-Bid Expression and Cytochrome c Release

To investigate Bid expression, Jurkat cells were treated with LV50 and then whole cell lysates were analyzed by Western blot, as described above, using goat anti-Bid antibodies (R&D Systems, Minneapolis, MN, USA). Furthermore, to evaluate cytochrome c release, we have isolated cytosolic proteins [51] from vehicle-treated Jurkat cells or cells incubated with LV50. Then, we performed a Western blot analysis, as described above, using mouse anti-cytochrome c antibodies (BD Biosciences, San Jose, CA, USA).

### 4.10. Statistical Analysis

Data are expressed as means ± standard deviation (SD) of at least three or more independent experiments. Statistical analysis was performed by parametric Student *t* test and *p* values of less than 0.05 were considered significant.

## Figures and Tables

**Table 1:**
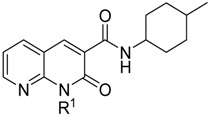
Radioligand binding data of CB91, LV58, LV62, and LV50 ^a^.

Compound	R^1^	K_i_ CB1R (nM)	K_i_ CB2R (nM)	K_i_ CB1R/K_i_ CB2R
CB91^26^	p–fluorobenzyl	200 ± 7.7	0.90 ± 0.038	222
LV62^27^	CH_2_(CH_2_)_4_OH	>10,000	3.60 ± 0.13	>187
LV58^27^	CH_2_(CH_2_)_3_F	1011 ± 46.5	1.36 ± 0.053	743
LV50	CH_2_(CH_2_)_3_Cl	224 ± 8.53	0.54 ± 0.017	415

^a^ Data represent mean values for at least three distinct assays made in duplicate and are reported as K_i_ (nM). Affinity of the tested compounds for cannabinoid receptor 1 (CB1R) and CB2R were assessed using membranes from HEK-293 cells transfected and [^3^H]CP55,940.

**Table 2:** Preliminary analysis of CB91, LV58, LV62, and LV50 ^a^.

Compound	Trypan Blue Positive Cells (%)	Viability (%)	subG1 Phase (%)
**24 h**			
CB91	2.10 ± 0.13	83.35 ± 7.34	2.35 ± 0.16
LV62	7.60 ± 0.55	86.75 ± 7.37	3.45 ± 0.27
LV58	2.85 ± 0.20	87.80 ± 7.26	2.41 ± 0.18
LV50	18.13 ± 1.26	77.78 ± 6.22	7.85 ± 0.60
**48 h**			
CB91	2.15 ± 0.16	78.80 ± 6.30	2.82 ± 0.17
LV62	10.5 ± 0.82	82.33 ± 6.25	8.88 ± 0.61
LV58	3.50 ± 0.22	85.50 ± 6.85	4.50 ± 0.26
LV50	23.93 ± 1.91	70.90 ± 5.31	18.7 ± 1.40
**72 h**			
CB91	2.30 ± 0.18	75.45 ± 4.35	3.93 ± 0.18
LV62	14.80 ± 0.88	70.25 ± 4.21	10.60 ± 0.53
LV58	3.8 ± 0.25	81.25 ± 4.46	5.19 ± 0.33
LV50	30.00 ± 2.05	59.32 ± 3.85	24.70 ± 1.95

^a^ Data represent mean ± SD of different experiments.

**Table 3:** Preliminary analysis of CB91, LV58, LV62, and LV50 after pretreatment with CB2R antagonist SR144528 ^a^.

Compound	Trypan Blue Positive Cells (%)	Viability (%)	subG1 Phase (%)
**24 h**			
SR144528 + CB91	2.00 ± 0.12	82.5 ± 7.30	2.31 ± 0.15
SR144528 + LV62	7.56 ± 0.54	85.73 ± 7.35	3.40 ± 0.26
SR144528 + LV58	2.8 ± 0.21	86.80 ± 7.15	2.39 ± 0.17
SR144528 + LV50	9.53 ± 0.86	87.68 ± 6.12	2.55 ± 0.20
**48 h**			
SR144528 + CB91	2.11 ± 0.16	77.50 ± 6.25	2.77 ± 0.17
SR144528 + LV62	10.10 ± 0.81	81.20 ± 6.20	8.87 ± 0.61
SR144528 + LV58	3.43 ± 0.21	84.00 ± 6.75	4.48 ± 0.25
SR144528 + LV50	12.50 ± 0.95	85.60 ± 6.81	12.70 ± 1.10
**72 h**			
SR144528 + CB91	2.31 ± 0.18	75.50 ± 4.35	3.92 ± 0.18
SR144528 + LV62	14.81 ± 0.87	70.24 ± 4.22	10.61 ± 0.53
SR144528 + LV58	3.79 ± 0.26	81.20 ± 4.45	5.18 ± 0.32
SR144528 + LV50	16.58 ± 1.25	86.18 ± 4.34	19.00 ± 2.05

^a^ Data represent mean ± SD of different experiments.

## Supplementary Materials

Supplementary materials can be found at http://www.mdpi.com/1422-0067/19/7/1958/s1.

## References

[1] Munro S., Thomas K.L., Abu-Shaar M. (1993). Molecular characterization of a peripheral receptor for cannabinoids. Nature.

[2] Fernandez-Ruiz J., Romero J., Velasco G., Tolón R.M., Ramos J.A., Guzmán M. (2007). Cannabinoid CB2 receptor: A new target for controlling neural cell survival?. Trends Pharmacol. Sci..

[3] Guzmán M., Duarte M.J., Blázquez C., Ravina J., Rosa M.C., Galve-Roperh I., Sánchez C., Velasco G., González-Feria L. (2006). A pilot clinical study of Δ9-tetrahydrocannabinol in patients with recurrent glioblastoma multiforme. Br. J. Cancer.

[4] Sarfaraz S., Adhami V.M., Syed D.N., Afaq F., Mukhtar H. (2008). Cannabinoids for cancer treatment: Progress and promise. Cancer Res..

[5] Velasco G., Sánchez C., Guzmán M. (2012). Towards the use of cannabinoids as antitumour agents. Nat. Rev. Cancer.

[6] Do Y., McKallip R.J., Nagarkatti M., Nagarkatti P.S. (2004). Activation through cannabinoid receptors 1 and 2 on dendritic cells triggers NF-kappaB-dependent apoptosis: Novel role for endogenous and exogenous cannabinoids in immunoregulation. J. Immunol..

[7] Galve-Roperh I., Sanchez C., Cortes M.L., Gómez del Pulgar T., Izquierdo M., Guzmán M. (2000). Anti-tumoral action of cannabinoids: Involvement of sustained ceramide accumulation and extracellular signal-regulated kinase activation. Nat. Med..

[8] Sanchez C., de Ceballos M.L., del Pulgar T.G., Rueda D., Corbacho C., Velasco G., Galve-Roperh I., Huffman J.W., Ramón y Cajal S., Guzmán M. (2001). Inhibition of glioma growth in vivo by selective activation of the CB(2) cannabinoid receptor. Cancer Res..

[9] Carracedo A., Gironella M., Lorente M., Garcia S., Guzmán M., Velasco G., Iovanna J.L. (2006). Cannabinoids induce apoptosis of pancreatic tumor cells via endoplasmic reticulum stress-related genes. Cancer Res..

[10] Casanova M.L., Blazquez C., Martinez-Palacio J., Villanueva C., Fernández-Aceñero M.J., Huffman J.W., Jorcano J.L., Guzmán M. (2003). Inhibition of skin tumor growth and angiogenesis in vivo by activation of cannabinoid receptors. J. Clin. Investig..

[11] Rueda D., Navarro B., Martinez-Serrano A., Guzman M., Galve-Roperh I. (2002). The endocannabinoid anandamide inhibits neuronal pro- genitor cell differentiation through attenuation of the Rap1/B-Raf/ERK pathway. J. Biol. Chem..

[12] Rueda D., Galve-Roperh I., Haro A., Guzman M. (2000). The CB1 cannabinoid receptor is coupled to the activation of c-Jun N-terminal kinase. Mol. Pharmacol..

[13] Greenhough A., Patsos H.A., Williams A.C., Paraskeva C. (2007). The cannabinoid Δ9-tetrahydrocannabinol inhibits RAS-MAPK and PI3K-AKT survival signalling and induces BAD-mediated apoptosis in colorectal cancer cells. Int. J. Cancer.

[14] Guzman M., Galve-Roperh I., Sanchez C. (2001). Ceramide: A new second messenger of cannabinoid action. Trends Pharmacol. Sci..

[15] Caffarel M.M., Moreno-Bueno G., Cerutti C., Palacios J., Guzman M., Mechta-Grigoriou F., Sanchez C. (2008). JunD is involved in the antiproliferative effect of Delta(9)-tetrahydrocannabinol on human breast cancer cells. Oncogene.

[16] Caffarel M.M., Sarrio D., Palacios J., Guzmán M., Sánchez C. (2006). Delta9-tetrahydrocannabinol inhibits cell cycle progression in human breast cancer cells through Cdc2 regulation. Cancer Res..

[17] Hohmann A.G. (2002). Spinal and peripheral mechanisms of cannabinoid antinociception: Behavioral, neurophysiological and neuroanatomical perspectives. Chem. Phys. Lipids.

[18] Karsak M., Gaffal E., Date R., Wang-Eckhardt L., Rehnelt J., Petrosino S., Starowicz K., Steuder R., Schlicker E., Cravatt B. (2007). Attenuation of allergic contact dermatitis through the endocannabinoid system. Science.

[19] Guindon J., Hohmann A.G. (2011). The endocannabinoid system and cancer: Therapeutic implication. Br. J. Pharmacol..

[20] Oesch S., Gertsch J. (2009). Cannabinoid receptor ligands as potential anticancer agents-high hopes for new therapies?. J. Pharm. Pharmacol..

[21] Izzo A.A., Camilleri M. (2008). Emerging role of cannabinoids in gastrointestinal and liver diseases: Basic and clinical aspects. Gut.

[22] Pacher P., Bátkai S., Kunos G. (2006). The endocannabinoid system as an emerging target of pharmacotherapy. Pharmacol. Rev..

[23] Benito C., Tolón R.M., Pazos M.R., Núñez E., Castillo A.I., Romero J. (2008). Cannabinoid CB2 receptors in human brain inflammation. Br. J. Pharmacol..

[24] Malan T.P.J., Ibrahim M.M., Lai J., Vanderah T.W., Makriyannis A., Porreca F. (2003). CB2 cannabinoid receptor agonists: Pain relief without psychoactive effects?. Curr. Opin. Pharmacol..

[25] Manera C., Cascio M.G., Benetti V., Allarà M., Tuccinardi T., Martinelli A., Saccomanni G., Vivoli E., Ghelardini C., Di Marzo V. (2007). New 1,8-naphthyridine and quinoline derivatives as CB2 selective agonists. Bioorg. Med. Chem. Lett..

[26] Manera C., Saccomanni G., Adinolfi B., Benetti V., Ligresti A., Cascio M.G., Tuccinardi T., Lucchesi V., Martinelli A., Nieri P. (2009). Rational design, synthesis, and pharmacological properties of new 1,8-naphthyridin-2(1*H*)-on-3-carboxamide derivatives as highly selective cannabinoid-2 receptor agonists. J. Med. Chem..

[27] Lucchesi V., Hurst D.P., Shore D.M., Bertini S., Ehrmann B.M., Allarà M., Lawrence L., Ligresti A., Minutolo F., Saccomanni G. (2014). CB2-selective cannabinoid receptor ligands: Synthesis, pharmacological evaluation, and molecular modeling investigation of 1,8-Naphthyridin-2(1*H*)-one-3-carboxamides. J. Med. Chem..

[28] Malfitano A.M., Laezza C., D’Alessandro A., Procaccini C., Saccomanni G., Tuccinardi T., Manera C., Macchia M., Matarese G., Gazzerro P. (2013). Effects on immune cells of a new 1,8-naphthyridin-2-one derivative and its analogues as selective CB2 agonists: IMPLICATIONS in multiple sclerosis. PLoS ONE.

[29] Malfitano A.M., Laezza C., Saccomanni G., Tuccinardi T., Manera C., Martinelli A., Ciaglia E., Pisanti S., Vitale M., Gazzerro P. (2013). Immune-modulation and properties of absorption and blood brain barrier permeability of 1,8-naphthyridine derivatives. J. Neuroimmune Pharm..

[30] Manera C., Saccomanni G., Malfitano A.M., Bertini S., Castelli F., Laezza C., Ligresti A., Lucchesi V., Tuccinardi T., Rizzolio F. (2012). Rational design, synthesis and anti-proliferative properties of new CB2 selective cannabinoid receptor ligands: An investigation of the 1,8-naphthyridin-2(1*H*)-one scaffold. Eur. J. Med. Chem..

[31] Ellert-Miklaszewska A., Kaminska B., Konarska L. (2005). Cannabinoids down-regulate PI3K/Akt and Erk signalling pathways and activate proapoptotic function of Bad protein. Cell Signal..

[32] McKallip R.J., Lombard C., Fisher M., Martin B.R., Ryu S., Grant S., Nagarkatti P.S., Nagarkatti M. (2002). Targeting CB2 cannabinoid receptors as a novel therapy to treat malignant lymphoblastic disease. Blood.

[33] Kroemer G., Galluzzi L., Brenner C. (2007). Mitochondrial membrane permeabilization in cell death. Physiol. Rev..

[34] Velasco G., Sánchez C., Guzmán M. (2016). Anticancer mechanisms of cannabinoids. Curr. Oncol..

[35] Cianchi F., Papucci L., Schiavone N., Lulli M., Magnelli L., Vinci M.C., Messerini L., Manera C., Ronconi E., Romagnani P. (2008). Cannabinoid receptor activation induces apoptosis through tumor necrosis factor alpha-mediated ceramide de novo synthesis in colon cancer cells. Clin. Cancer Res..

[36] Capozzi A., Mantuano E., Matarrese P., Saccomanni G., Manera C., Mattei V., Gambardella L., Malorni W., Sorice M., Misasi R. (2012). A new 4-phenyl-1,8-naphthyridine derivative affects carcinoma cell proliferation by impairing cell cycle progression and inducing apoptosis. Anticancer Agents Med. Chem..

[37] Blazquez C., Carracedo A., Barrado L., Real P.J., Fernández-Luna J.L., Velasco G., Malumbres M., Guzmán M. (2006). Cannabinoid receptors as novel targets for the treatment of melanoma. FASEB J..

[38] Blazquez C., Gonzalez-Feria L., Alvarez L., Haro A., Casanova M.L., Guzmán M. (2004). Cannabinoids inhibit the vascular endothelial growth factor pathway in gliomas. Cancer Res..

[39] Sarfaraz S., Afaq F., Adhami V.M., Malik A., Mukhtar H. (2006). Cannabinoid receptor agonist-induced apoptosis of human prostate cancer cells LNCaP proceeds through sustained activation of erk1/2 leading to G1 cell cycle arrest. J. Biol. Chem..

[40] Gustafsson K., Christensson B., Sander B., Flygare J. (2006). Cannabinoid Receptor-Mediated Apoptosis Induced by R(+)-Methanandamide and Win55,212-2 Is Associated with Ceramide Accumulation and p38 Activation in Mantle Cell Lymphoma. Mol. Pharmacol..

[41] Munson A.E., Harris L.S., Friedman M.A., Dewey W.L., Carchman R.A. (1975). Antineoplastic activity of cannabinoids. J. Natl. Cancer Inst..

[42] Costa M.A., Fonseca M.B., Teixeira N.A., Correia-da-Silva G. (2015). The endocannabinoid anandamide induces apoptosis in cytotrophoblast cells: Involvement of both mithocondrial and death receptor pathways. Placenta..

[43] Herrera B., Carracedo A., Diez-Zaera M., Gómez del Pulgar T., Guzmán M., Velasco G. (2006). The CB2 cannabinoid receptor signals apoptosis via ceramide-dependent activation of the mitochondrial intrinsic pathway. Exp. Cell Res..

[44] Jia W., Hegde V.L., Singh N.P., Sisco D., Grant S., Nagarkatti M., Nagarkatti P.S. (2006). Delta9-Tetrahydrocannabinol-Induced Apoptosis in Jurkat Leukemia T Cells Is Regulated by Translocation of Bad to Mitochondria. Mol. Cancer Res..

[45] Guzmán M. (2003). Cannabinoids: Potential anticancer agents. Nat. Rev. Cancer.

[46] Gatley S.J., Lan R., Pyatt B., Gifford A.N., Volkow N.D., Makriyannis A. (1997). Binding of the non-classical cannabinoid CP 55,940, and the diarylpyrazole AM251 to rodent brain cannabinoid receptors. Life Sci..

[47] Huffman J.W., Zengin G., Wu M.J., Lu J., Hynd G., Bushell K., Thompson A.L., Bushell S., Tartal C., Hurst D.P. (2005). Structure-activity relationships for 1-alkyl-3-(1-naphthoyl)indoles at the cannabinoid CB(1) and CB(2) receptors: Steric and electronic effects of naphthoyl substituents. New highly selective CB(2) receptor agonists. Bioorg. Med. Chem. Lett..

[48] Cheng Y.C., Prusoff W.H. (1973). Relationship between the inhibition constant (K1) and the concentration of inhibitor which causes 50 per cent inhibition (I50) of an enzymatic reaction. Biochem. Pharmacol..

[49] Wilson A.P., Freshney R.I. (1992). Citotoxicity and viability assays. Animal Cell Culture: A Practical Approach.

[50] Sorice M., Matarrese P., Tinari A., Giammarioli A.M., Garofalo T., Manganelli V., Ciarlo L., Gambardella L., Maccari G., Botta M. (2009). Raft component GD3 associates with tubulin following CD95/Fas ligation. FASEB J..

[51] Degli Esposti M., Matarrese P., Tinari A., Longo A., Recalchi S., Khosravi-Far R., Malorni W., Misasi R., Garofalo T., Sorice M. (2017). Changes in membrane lipids drive increased endocytosis following Fas ligation. Apoptosis.

