# Supplementary material for: Anti-Proliferative Properties and Proapoptotic Function of New CB2 Selective Cannabinoid Receptor Agonist in Jurkat Leukemia Cells
Source: Int J Mol Sci. 2018 Jul 4;19(7):1958. doi: 10.3390/ijms19071958 (PMC6073364; doi:10.3390/ijms19071958)
Supplement: Supplementary file 1 [file ijms-19-01958-s001.pdf]

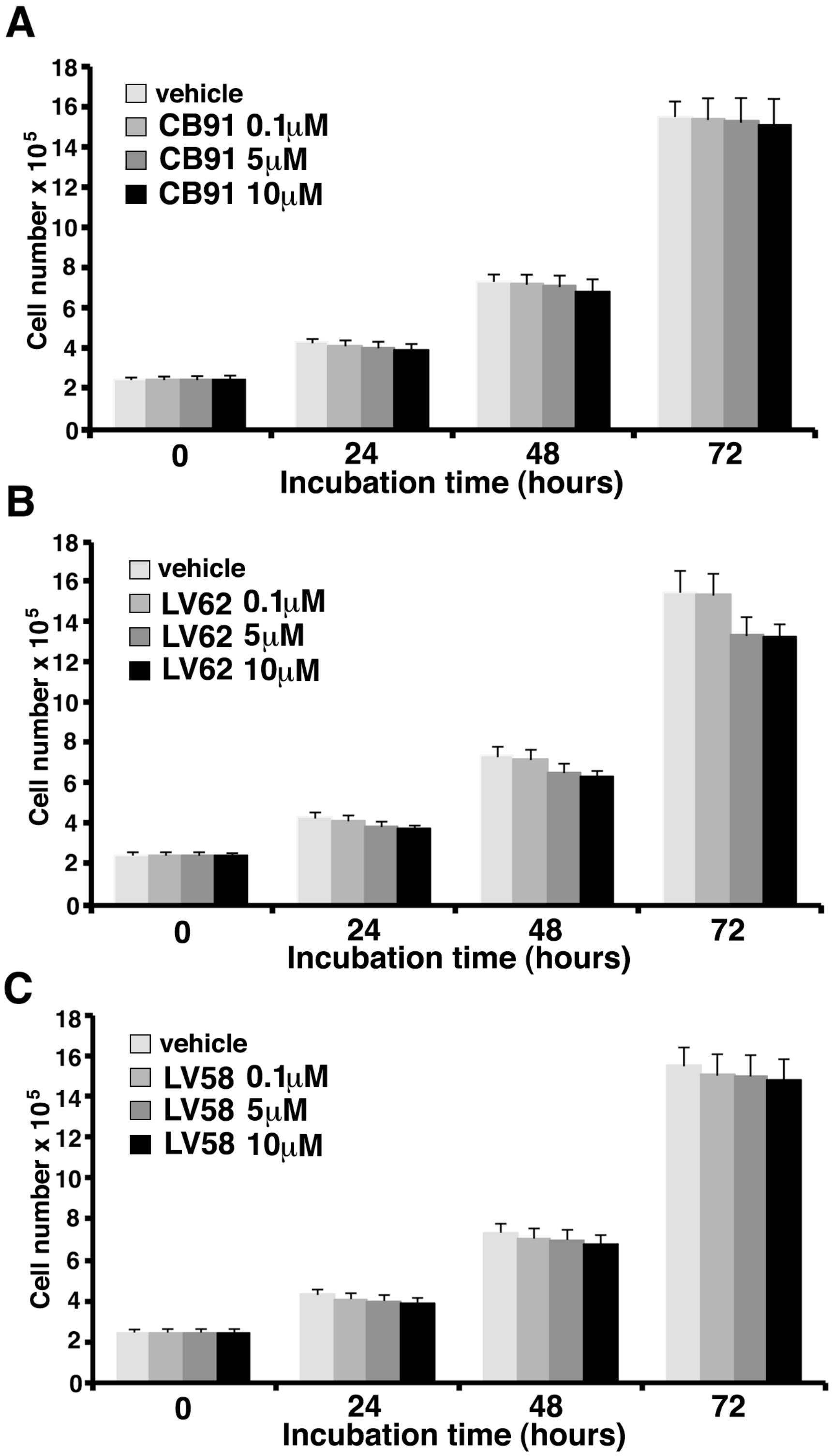

Figure S1. Trypan Blue exclusion test: CB91, LV62, and LV58 effect on cell viability. Jurkat cells were cultured with three different concentrations of the compound (0.1μM, 5 μM, and 10 μM) for 24, 48, and 72 h. The number of viable cells was determined by Trypan Blue exclusion test. Data are reported as the mean ± SD among ten independent experiments. Statistical analysis indicated: treated versus vehicle. NS, not significant.
